# ROCK and PDE-5 Inhibitors for the Treatment of Dementia: Literature Review and Meta-Analysis

**DOI:** 10.3390/biomedicines10061348

**Published:** 2022-06-08

**Authors:** Dong-Hun Lee, Ji Young Lee, Dong-Yong Hong, Eun Chae Lee, Sang-Won Park, Yu Na Jo, Yu Jin Park, Jae Young Cho, Yoo Jin Cho, Su Hyun Chae, Man Ryul Lee, Jae Sang Oh

**Affiliations:** 1 Department of Neurosurgery, College of Medicine, Soonchunhyang University, Cheonan Hospital, Cheonan 31151, Korea; madeby58@gmail.com (D.-H.L.); applesori82@gmail.com (J.Y.L.); dydehdghd@gmail.com (D.-Y.H.); lec9589@gmail.com (E.C.L.); ppphilio3@gmail.com (S.-W.P.); 2 Soonchunhyang Institute of Medi-bio Science (SIMS), Soon Chun Hyang University, Cheonan 31151, Korea; 3 Department of Medicine, College of Medicine, Soonchunhyang University, Cheonan 31151, Korea; persil0507@naver.com (Y.N.J.); park.yj0910@gmail.com (Y.J.P.); chojy9808@gmail.com (J.Y.C.); whro0621@naver.com (Y.J.C.); chaesuhyun2000@naver.com (S.H.C.)

**Keywords:** Alzheimer’s disease, cGMP, ERM, LIMK, meta-analysis, MLC, Morris water maze, PDE-5 inhibitor, ROCK inhibitor, vascular dementia

## Abstract

Dementia is a disease in which memory, thought, and behavior-related disorders progress gradually due to brain damage caused by injury or disease. It is mainly caused by Alzheimer’s disease or vascular dementia and several other risk factors, including genetic factors. It is difficult to treat as its incidence continues to increase worldwide. Many studies have been performed concerning the treatment of this condition. Rho-associated kinase (ROCK) and phosphodiesterase-5 (PDE-5) are attracting attention as pharmacological treatments to improve the symptoms. This review discusses how ROCK and PDE-5 affect Alzheimer’s disease, vascular restructuring, and exacerbation of neuroinflammation, and how their inhibition helps improve cognitive function. In addition, the results of the animal behavior analysis experiments utilizing the Morris water maze were compared through meta-analysis to analyze the effects of ROCK inhibitors and PDE-5 inhibitors on cognitive function. According to the selection criteria, 997 publications on ROCK and 1772 publications on PDE-5 were screened, and conclusions were drawn through meta-analysis. Both inhibitors showed good improvement in cognitive function tests, and what is expected of the synergy effect of the two drugs was confirmed in this review.

## 1. Introduction

Dementia refers to a significant decrease in an individual’s cognitive level accompanied by memory, thought, and behavior-related disorders caused by brain damage due to injury or disease, and it hinders the patient’s social function [1]. Although dementia usually occurs in the elderly, it is not an inevitable consequence of aging. It is currently the seventh leading cause of death among all diseases and a major cause of disability among the elderly worldwide. Dementia has a physical, psychological, social, and economic impact, not only on patients with dementia but also on their acquaintances, families, and society at large.

There are two main types of dementia: Alzheimer’s disease (AD) and vascular dementia (VD). AD is a neurodegenerative disease and accounts for 60–70% of patients with dementia [2]. AD causes abnormal accumulation of amyloid-beta (Aβ) as amyloid plaque and tau protein and the formation of neurofibrillary tangles (NFT), resulting in gradual loss of brain function [3]. In the early stages, patients experience mild cognitive impairment, indifference, and depression [4]. As the disease progresses, disturbances in language, execution skills, short-term memory, and long-term memory become more pronounced; in the latter stages of severe illness, daily life activities become difficult without dependence on caregivers [5,6,7,8,9,10].

The other type of dementia, VD, is caused due to issues with the brain blood supply, which usually worsens cognitive performance and gradually decreases due to minor strokes [11]. VD also exhibits mild cognitive impairment, acute or subacute progressive cognitive impairment, depending on severity, and is often accompanied by AD [12].

Currently, more than 55 million people worldwide live with dementia, and nearly 10 million new patients are identified every year. In 2020, the prevalence of AD in the United States was estimated at 5.3% in the 60–74 year age group, 13.8% in the 74–84 year age group, and 34.6% in the ≥85 year age group [13]. The prevalence of VD in the United States was 2.43% in all patients > 71 years, and it was found that it doubles every 5.1 years [14,15]. In addition, dementia caregivers spend an average of 47 h per week on patient care, with direct and indirect costs of caring for patients ranging from $18,000 to $77,500 in the United States [16].

Several considerations have been proposed to treat AD [17]. The goal of treatment for AD patients is to improve the loss of memory and cognition, or slow the loss as much as possible. Anticholinesterases, such as Donepezil, attempted to improve AD by increasing cholinergic synaptic transmission in synaptic gaps. As an antioxidant treatment to protect neurons from oxidative stress, there is also a method of slowing the late-stage progression of AD by administering Alpha-tocopherol (vitamin E) and Idebenone. The most recently FDA-approved AD drug, aducanumab, is a monoclonal antibody that targets AB to reduce the accumulation of AB. However, there is still controversy over the instability [18,19].

## 2. Pathophysiology of Dementia

AD is characterized by NFTs of extracellular Aβ plaque and highly phosphorylated tau [20]. According to the amyloid hypothesis, extracellular Aβ deposition is the underlying cause of AD. It is supported by the fact that patients with disorders in expressing the gene for amyloid precursor protein (APP) exhibit early Alzheimer’s symptoms [21,22,23]. APP is a transmembrane glycoprotein that is the precursor of amyloid β (Aβ), a 40–42 amino acid peptide that is the principal constituent of senile plaques and cerebrovascular deposits in AD [24,25,26,27]. The Aβ is toxic to neurons and can cause neurodegenerative mechanisms. The tau hypothesis explains that tau protein abnormalities cause the initiation of the disease. Highly phosphorylated tau forms an NFT inside the nerve cell body, decomposes the microtubules, and causes the collapse of the neuron’s transport system along with the destruction of the cell skeleton [28,29]. Another hypothesis suggests that neuroinflammation causes AD, which has been discussed intensively [30,31]. The accumulation of Aβ and NFT increases the expression of microglia and astrocyte, which are glial cells responsible for nerve immunity in the brain, which, after exposure to amyloid-beta, secretes cytokine, interleukin, nitric oxide, and other cytotoxic reactions in patients, exacerbate AD [31,32,33].

VD is associated with several cerebrovascular risk factors [34]. Some factors involved in cerebrovascular disease include sex, age, vascular risk factors, some disorders, genetic factors, and inflammation. The representative diseases include arteriosclerosis, cerebral amyloid angiopathy, cerebral autosomal dominant arteriopathy with subcortical infarcts and leukoencephalopathy, basal ganglia calcification, and other intracerebral vasculopathies. Other risk factors include stroke, psychological stress or life history, and fat diet intake. Additionally, several small thromboembolic strokes or strokes at major locations, such as the frontal lobe, sagittal, or temporal lobe, can cause cognitive disorders.

A mechanism of ischemic VD involves a major vascular disease in which blood flow in the brain decreases because of arterial stenosis. However, this has not been sufficiently investigated clinically, and the large vessel disease associated with VD is unclear. However, small changes in blood vessels cause damage to brain tissue and are potentially responsible for cognitive impairment. Another possible mechanism influencing small blood vessel changes is incomplete ischemia and selective tissue necrosis, causing selective neurological necrosis due to decreased functioning of neuroglial cells and microvessels [35,36,37].

## 3. Rho-Associated Protein Kinase (ROCK) and Dementia

ROCK is the downstream effector protein of RhoA, a GTP-binding protein [38,39]. It belongs to the AGC (PKA/PKG/PKC) family of serine-threonine-specific protein kinase. It plays an important role in vasoconstriction, and its isoforms include ROCK1 and ROCK2 [39,40,41,42]. ROCK1 is commonly expressed in all tissues but less in the brain and skeletal muscles. ROCK2 is more abundant in the brain, muscles, heart, lungs, and placenta [43,44,45,46,47]. ROCK is related to hypoxia exposure, endothelial dysfunction, vascular smooth muscle cell (VSMC) proliferation, reactive oxygen species (ROS), and inflammatory cell migration. It is the main regulator of actin organization [48,49]. It phosphorylates various substrates, including Lin-11/Isl-1/Meg-3 (LIM) kinase (LIMK), Myosin light chain (MLC), and MLC phosphatase (MLCP). ROCK influences amyloid-beta production, NFT formation, and neuroinflammatory regulation, affecting AD incidence [50,51,52,53].

### 3.1. ROCK and AD

Mutation of presenilin (PSEN) was confirmed in cases of early-onset AD [54]. The APP is cleaved by both β-secretases and γ-secretase enzymes to form Aβ, and PSEN is a component of γ secretase. Therefore, mutation of PSEN leads to an increase in the Aβ42:Aβ40 ratio due to an increase in the expression of Aβ42, which promotes early Aβ deposition [55]. ROCK contributes to these secretases cleaving APP to increase Aβ production. The increase in Aβ levels further suggests a positive feedback role for ROCK, though the specific basic mechanism for this remains unclear [56].

In addition, hyperphosphorylation of tau, a characteristic of AD, seems to be associated with ROCK. Although no specific mechanism has been identified, ROCK activation activates tau kinase and inhibits tau phosphatase, increasing the expression of P-tau and oligomeric tau. It also reduces the microtubule-binding of tau and increases the formation of NFTs in neurons [57].

### 3.2. ROCK and Vascular Remodeling

**Figure 1:**
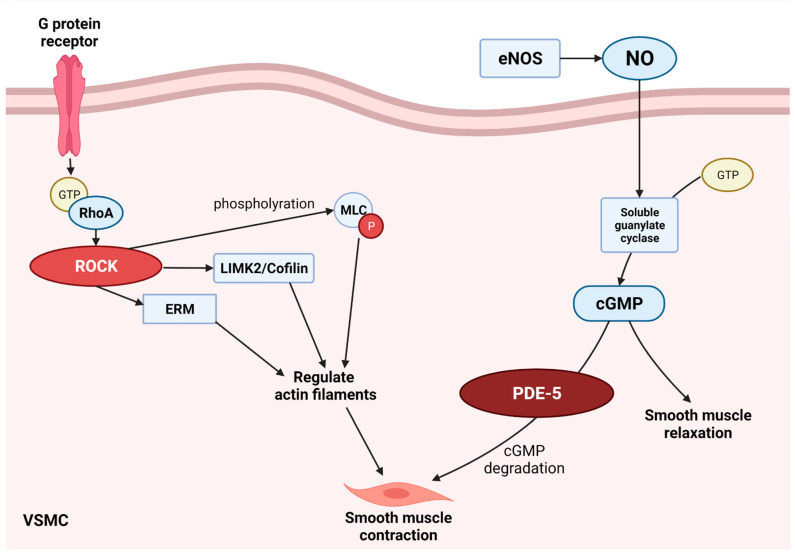
The RhoA/ROCK pathway and PDE-5 pathway contribute to smooth muscle contraction. ROCK prevents dephosphorylation of phosphorylated MLC and contributes to smooth muscle contraction by activating the LIMK2/cofilin pathway and the ERM pathway. PDE-5 decomposes increased cGMP from eNOS/NO, contributing to the contraction of VSMC. cGMP: cyclic Guanosine monophosphate; eNOS: endothelial nitric oxide synthase; ERM: ezrin/radixin/moesin; GTP: guanosine triphosphate; LIMK: Lin11-Isl1-Mec3 kinase; MLC: myosin light chain; NO: nitric oxide; PDE-5: phosphodiesterase-5; ROCK: rho-associated protein kinase.

ROCK is involved in vascular remodeling, which starts with RhoA. Downstream targets of ROCK include MLCP, LIMK, Ezrin/Radixin/Moesin (ERM) intermediate filaments, and other factors affecting intracellular processes that are important for cell contraction, movement, proliferation, and morphology (Figure 1). RhoA binds to GTP from the G-protein-receptor. When the cytoplasmic concentration of ROCK and Ca^2+^ increases through guanine nucleotide exchange factors, the increased ROCK phosphorylates MLC [58]. The increase in MLC phosphorylation results in the contraction of the smooth muscle by combining myosin crossbridge and actin filaments. LIM phosphorylated by ROCK then phosphorylates cofilin to inhibit actin decomposition activity. Cofilin is an actin-binding protein associated with the rapid depolymerization of actin microfibers. It regulates the assembly and decomposition of actin filaments. The ERM family crosslinks the protoplasmic membrane and the actin filament to prevent actin-binding according to the folding of the ERM protein.

ROCK is also related to endothelial NOS (eNOS): it is the upstream negative regulator of eNOS, and its expression reduces eNOS expression [59]. eNOS has a protective function in the cardiovascular system due to nitric oxide (NO) production. NO catalyzes the conversion of guanosine triphosphate to cGMP by activating the enzyme soluble guanylate cyclase (sGC). This cGMP acts on vascular relaxation and contributes to vascular remodeling. NO production of eNOS inhibited by ROCK ultimately reduces the cGMP of VSMCs, thereby constricting blood vessels. In animal experiments of the VD model, an increase in ROCK expression was shown [60].

### 3.3. ROCK and Neuroinflammation

Neuroinflammation is an inflammation of nerve tissue and can begin as a response to various signals, including infection, traumatic brain damage, toxic metabolites, or autoimmune. Neuroinflammation is a common feature observed in many neurodegenerative disorders and is an important factor in neurodegenerative progression. The involvement of local innate immune responses contributes greatly to central nervous system (CNS) damage [61].

**Figure 2:**
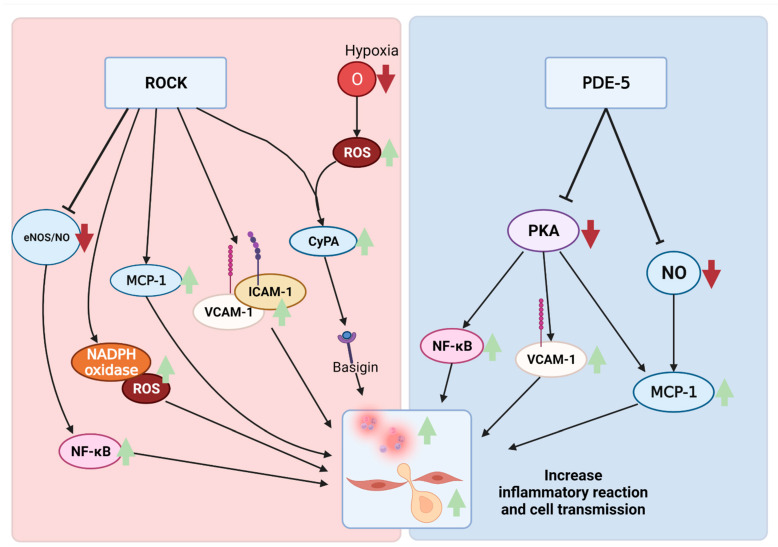
Improvement of the inflammatory response and recruitment of inflammatory cells due to various factors in the ROCK and PDE family. CyPA: cyclophilin A; ICAM-1: intercellular adhesion molecule 1; MCP-1: monocyte chemoattractant protein 1; NF-κB: nuclear factor kappa-light-chain-enhancer of activated B cells; NO: nitric oxide; PDE-5: phosphodiesterase-5; PKA: protein kinase A; ROCK: rho-associated protein kinase; ROS: reactive oxygen species; VCAM-1: vascular cell adhesion molecule 1.

Activation of the RhoA/ROCK pathway increases the permeability of inflammatory factors in response to inflammatory stimuli (Figure 2). The RhoA/ROCK pathway, through G-protein-receptors, is upregulated by chemokine-like MCP-1 and increases resolution from occludin, claudin-5, ZO-1 Ser/Thr phosphorylation, and tight junctions (TJ) to increase the barrier [62]. In addition, intracellular adhesion molecule 1 (ICAM-1) and vascular cell adhesion protein 1 (VCAM-1) increase inflammatory cell penetration in the CNS from RhoA/ROCK activation. Studies have shown that ROCK activation promotes neutrophil penetration in inflammation through NADPH oxidase activation and ROS generation.

ROCK also directly increases the inflammatory response. Section 3.2 mentions eNOS and NO, which also inhibit chemokine and nuclear factor kappa B (NF-κB). Therefore, an increase in ROCK leads to a decrease in eNOS, resulting in an increase in the inflammatory response. In addition, the activated RhoA induces activation of p38 mitogen-activated protein kinase (p38 MAPK), which induces up-regulation of IL-4, IL-10, and INF-γ production [63,64]. ROCK2 also serves to phosphorylate the transcription factor IRF4 required for IL-17 and IL-21 generation and IL-17 T cell differentiation [65]. A study has shown that upon applying ischemic injury to the brain, the microglial proliferation contributing to neuroinflammation increases [66].

Cyclophilin A (CyPA) is a protein belonging to the immunophilin family. When ROS is induced in a hypoxic state, it stimulates CyPA secretion along with ROCK activity. CyPA secreted from VSMCs binds to basigin, a receptor outside the cell, to regulate the cell signal pathway [67] and acts as a chemical inducer for inflammatory cells [68,69].

## 4. Phosphodiesterase-5 (PDE-5) and Dementia

The PDE superfamily consists of 11 subtypes, PDE1-PDE11, and classification is based on sequence homogeneity [70]. PDE-1, PDE-2, PDE-3, PDE-10, and PDE-11 hydrolyze cGMP and cAMP; PDE-4, PDE-7, and PDE-8 preferentially cleave cAMP; and PDE-5, PDE-6, and PDE-9 preferentially cleave cGMP. PDE- 5 is an enzyme that hydrolyzes cyclic nucleotides, cAMP, and cGMP. cAMP is used for intracellular signaling to deliver cAMP-dependent pathways. cGMP regulates ion channels, glycogen degradation, and apoptosis and relaxes vascular smooth muscle tissue. In addition, the cGMP signaling pathway regulates several psychological processes, including vascular tension, visual signal transmission, energy metabolism, kidney function, bowel movement, fat decomposition, oocyte maturation, cerebellar motion regulation, transcription, cell growth, and anti-inflammatory function [71,72,73,74,75,76,77,78,79].

### 4.1. PDE-5 and AD

The relationship between PDE-5 and AD focuses on the NO pathway. The NO/sGC/cGMP signaling pathway appears abnormally in the AD brain [80,81,82,83]. In aging wild mice, eNOS deficiency showed an increase in Aβ production [84,85], and the depletion of iNOS in AD mice with APP mutation resulted in elevated levels of Aβ and hyperphosphorylation of tau [86]. This hyperphosphorylation of tau is thought to be because Akt inhibits glycogen synthase kinase-3 beta (GSK-3β), mediating tau phosphorylation due to the activation of the P13K/Akt pathway of NO. Therefore, the decrease in NO due to PDE-5 leads to an increase in tau phosphorylation. PDE-5 is upregulated in the cerebrospinal fluid (CSF) of patients with AD, and cGMP levels are decreased [87].

### 4.2. PDE-5 and Vascular Remodeling

PDE-5 breaks down cGMP produced from NO/sGC/GTP signals. cGMP acts on blood vessels and contributes to vascular relaxation. When the cGMP in the VSMCs is reduced by PDE-5, the blood flow to the brain may reduce due to vasoconstriction (Figure 1).

### 4.3. PDE-5 and Neuroinflammation

cGMP regulates intracellular inflammatory responses. Monocyte chemoattractant protein-1 (MCP-1) is a chemokine that contributes to the inflammatory response by recruiting monocyte, memory cells, and dendritic cells at the inflammatory site [82,88]. NO reduces the expression of MCP-1 mRNA, and the reduction of NO increases the expression of MCP-1 mRNA [89]. The regulation of the expression of MCP-1 may reduce the decomposition of IκB inhibiting NF-κB, thereby reducing the inflammatory reaction. PDE-5 may decompose cGMP in the initial steps of this process, contributing to neuroinflammation.

Astrocyte is a neuroglial cell of the brain and spinal cord. It is involved in blood-brain barrier formation and function [90], neurotransmission [91], nutrition to nerve tissue, the balance of extracellular ions, and regulation of cerebral blood flow [92]. The central immune role of astrocytes is controlled through the cGMP/PKG pathway through NO [93]. Protein kinase G (PKG) phosphorylates many targets by cGMP and is involved in functions such as smooth muscle relaxation. cGMP inhibits the expression of major histocompatibility complex II (MHC-II) derived from interferon-γ (INF-γ) in astrocytes and the expression of matrix metallopeptidase 9 (MMP-9) and tumor necrosis factor-α (TNF-α) induced by lipopolysaccharide (LPS) [93,94]. PDE-5 can lead to neuroinflammation by increasing the expression of INF-α, MMP-9, and TNF-α derived from astrocytes in the brain by decomposing cGMP.

## 5. Inhibition of ROCK and PDE-5 Pathway for Neuroprotection

Overexpression of ROCK has been shown to cause an increased inflammatory response, increased oxidative stress, high oxidation of tau, and cognitive decline due to β-amyloid accumulation. In such a situation, ROCK inhibitors are a good choice for treating dementia. Fasudil is a typical ROCK inhibitor with potential neuroprotective effects that can cause neurogenesis and increased neuronal viability [95,96]. Inhibition of ROCK2 leads to nerve survival and axon stability [95,97]. Like Fasudil, a representative ROCK inhibitor, Y-27632 is a good option for dementia treatment. The resulting ROCK inhibition has been shown to reduce TNF-α mediated monocyte migration [98,99]. ROCK-suppressed macrophages showed reduced chemotaxis for MCP-1/CCL2.

PDE-5 suppression is another possibility to improve dementia. PDE-5 inhibitors increase the cGMP concentration by blocking the cGMP decomposition of PDE-5 described above, and the increased cGMP expands blood vessels and improves blood flow through smooth muscle tissue relaxation. Typical PDE-5 inhibitors include sildenafil, vardenafil, and tadalafil. Subsequent animal studies with sildenafil demonstrated long-term retention of an inhibitory avoidance response in mice. In an in vitro study using N9 microglia, it was shown that cGMP accumulated because of regression of PDE-5 following sildenafil treatment could contribute to inhibiting microglia activation. In addition, injection of PDE-5 inhibitors into the ischemic stroke rat model with reduced cognitive function through middle cerebral artery occlusion (MCAo) improved neurological deficits and anxiogenic disorder and improved locomotion [100].

## 6. Meta-Analysis of ROCK Inhibitors and PDE-5 Inhibitors in Animal Experiments

In this study, a meta-analysis was performed to investigate the relationship between cerebrovascular disease and drug effects of ROCK and PDE-5 inhibition in animal models. A meta-analysis is a quantitative, formal, epidemiological study design used to systematically assess previous research studies to derive conclusions about that body of research. It can be performed when there are several scientific studies addressing the same problems, and each study reports measurements that are expected to be somewhat error-prone. The efficacy of ROCK inhibitors and PDE-5 inhibitors as drug treatments for dementia in animal models is analyzed and presented here.

### 6.1. Methods

#### 6.1.1. Search Strategy and Selection Criteria

Multiple comprehensive databases, such as PubMed, EMBASE, and the Cochrane Library, were used to search studies on ROCK inhibitors and PDE-5 inhibitors. The search was conducted on all manuscripts published so far without restriction on the year of publication. MeSH keywords were searched and are specified in the attached supplementary data: Keywords for ROCK inhibitors and PDE-5 inhibitors. The searched publications were evaluated for quality using the Newcastle-Ottawa scale (Table 1), and data were extracted from each study after completing the search.

#### 6.1.2. Data Extraction

The Morris water navigation task, also known as the Morris water maze (MWM), is a behavioral technique mostly used with rodents [124]. It is predominantly used in behavioral neuroscience to study spatial learning and memory. The basic procedure involved in MWM is that the mouse or rat is placed in a large circular pool and must find an invisible or visible platform that allows it to escape the water using various cues. The time it takes to escape is measured, and the maze is divided into quarters to help measure how long the animal stays in the target area. Animal models of neurotrauma, cerebrovascular disease, developmental disorders, metabolic disorders, AD, and other disorders with neurocognitive disorders and cognitive complications have been demonstrated to differ from healthy models using MWM [125,126,127,128,129,130,131,132,133,134,135,136,137,138,139,140]. In addition, as an evaluation of neurocognitive treatment, it was confirmed that the performance of MWM improved following behavior, pharmacological, and neurosurgical interventions [141,142,143,144,145,146].

Two reviewers (D.H.L. and J.S.O.) independently extracted data according to a predetermined data extraction form. Duplicate data was removed using Endnote. Features extracted from each study include the first author’s name, publication year, pathology induction method of the disease group, drugs used in the experimental group receiving pharmacotherapy, gender, age, pharmacotherapy method, drug dosage, disease classification (AD or VD), MWM—mean value of time spent in the target quadrant (TSTQ), SD values (%), and mice participating in the experiment (Table 2 and Table 3).

#### 6.1.3. Data Analysis

Meta-analysis was performed using a random effect model. The results were presented according to the 95% confidence interval (CI). A heterogeneity test was performed using the Cochran Q test, and a publication bias test was performed using the Egger’s test. Statistical analysis was conducted using Revman (version 5) software.

Because of the high heterogeneity, each group was sub-classified into a subgroup, and a meta-analysis was performed.

### 6.2. Results

**Figure 3:**
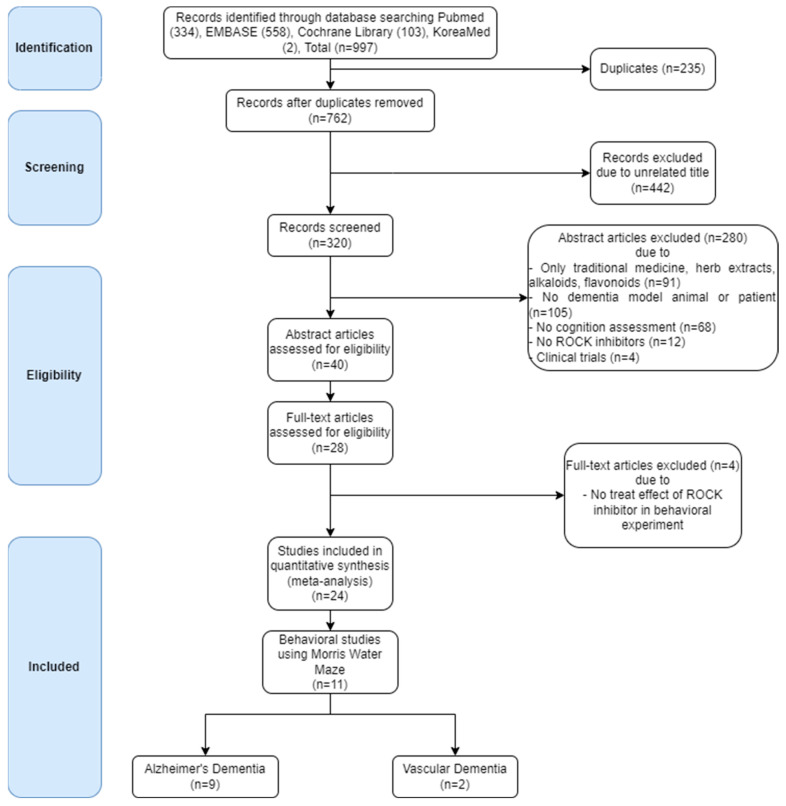
The ROCK inhibitor study was conducted independently by two reviewers according to the screening criteria. The studies were selected in the following order: (1) Duplicate articles were excluded. (2) Those with unrelated titles were excluded. Subsequently, (3) those with unrelated abstracts were excluded, and the exclusion considerations for the abstracts were as follows: only traditional medicine, herb extracts, alkaloids, and flavonoids. No dementia model animal or patient. No cognition assessment. No ROCK inhibitors. No clinical trials. (4) The following text was checked to exclude articles that did not study the effect of ROCK inhibitors on behavioral experiments. (5) Studies included in the quantitative synthesis. (6) Finally, only studies using the Morris water maze were left.

**Figure 4:**
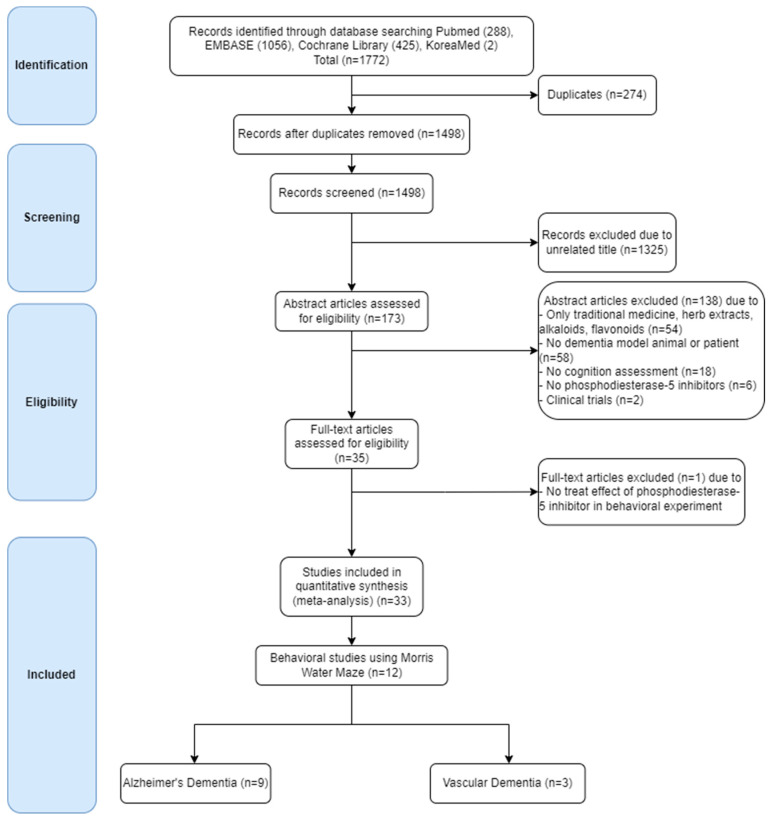
The phosphodiesterase-5 inhibitor study was performed independently along with the flow chart by two reviewers: (1) Duplicate articles were excluded. (2) Publications with unrelated titles and abstracts were excluded. (3) According to the eligibility, publications that had no therapeutic effect in the behavioral experiment were excluded. (4) Full-text articles that were not available for meta-analysis, that had no animal, cognition, or Morris water maze test, were excluded.

A total of 997 publications on ROCK inhibitors were identified through the search formula. According to the screening criteria, 11 publications were finally selected (Figure 3) after (1) removing duplicates; (2) removing articles with an irrelevant title; (3) removing articles with an irrelevant abstract (excluded if it was based only traditional medicine, herb extracts, alkaloids, flavonoids, no dementia model animal or patient, no cognition assessment, no ROCK inhibitors, or there was no clinical trials); (4) evaluating the abstract and full text for eligibility; (5) excluding articles that effect of ROCK inhibitors that failed to demonstrate any effects in behavior test; and (6) articles containing quantitative data of TSTQ of MWM test for meta-analysis. Of these, nine studies were on AD and two on VD. For PDE-5 inhibitors, 1772 publications were searched and screened according to the screening criteria (Figure 4). Finally, 12 publications were selected according to the screening criteria. Nine of these focused on AD and three on VD. The date of the most recent publication was 31 July 2021 [147].

A total of 414 mice participated in the 23 finally selected studies. Both male and female mice were included in these studies. Their age was widely distributed from 8 weeks to 18 months (Table 2).

Of these, 330 (79.8%) mice had AD, and 84 (20.2%) mice had VD. Methods that induced AD included drug administration and genetic mutations. The intracerebroventricular streptozotocin (ICV-STZ) method, which induces AD in animals without transformation, shows mitochondrial abnormalities [148], decreased glucose use, increased tau phosphorylation, and neurochemical changes in the brain, such as the 3xTG-AD mouse [149]. AD was induced in 64 (19.4%) mice through ICV-STZ injection. In addition to drug induction, there is a model that mimics human AD with genetic mutations. Further, 235 (71.2%) mice developed AD due to gene mutations. APP is a precursor molecule that produces Aβ and is a major component of amyloid plaque found in the brain of AD patients. PSEN-1 (PS1) plays an important role in Aβ generation by cleaving APPs and regulating their activity. Tg2576 and J20 induced mutations in these APPs, and APP/PS1 induced mutations in both APP and PS1 resulted in AD.

Mice with conditions imitating VD-induced arteriosclerosis with cholesterol crystals, ischemic stroke with bilateral common carotid artery occlusion (BCAO), or bilateral common carotid artery ligation had hypoxic damage to the brain.

ROCK inhibitors for pharmacological treatment of dementia (11 out of 23) include Fasudil and Y-27632. *Ganoderma lucidum* triterpenoids, *Edaravone*, and *Clitoria ternatea* have also been verified to inhibit the ROCK pathway. PDE-5 inhibitors (12 out of 23), including sildenafil, tadalafil, vardenafil, CM-414, and KJH-1002 have also been verified in each publication. The drugs were either stereotaxic, orally-administered, injected into the left lateral ventricle, administered through gavage, or through intraperitoneal or intracerebral vascular injection. Of all mice, a total of 205 (49.5%) mice were treated with either ROCK inhibitors or PDE-5 inhibitors, 98 (23.7%) of which were treated with ROCK inhibitors, and 107 (25.8%) were treated with PDE-5 inhibitors.

**Figure 5:**
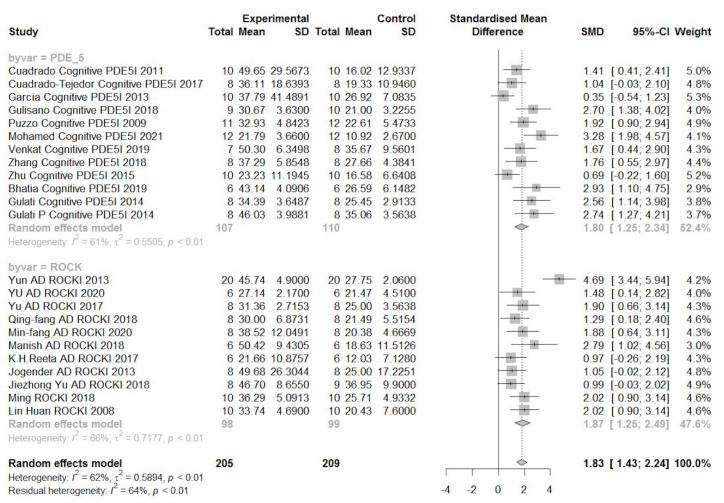
This forest plot shows an analysis of the difference between the experimental group and the control group of each subgroup by standardized mean difference (SMD). Because of the high heterogeneity, each group was analyzed by dividing it into subgroups. SD: standard deviation.

**Figure 6:**
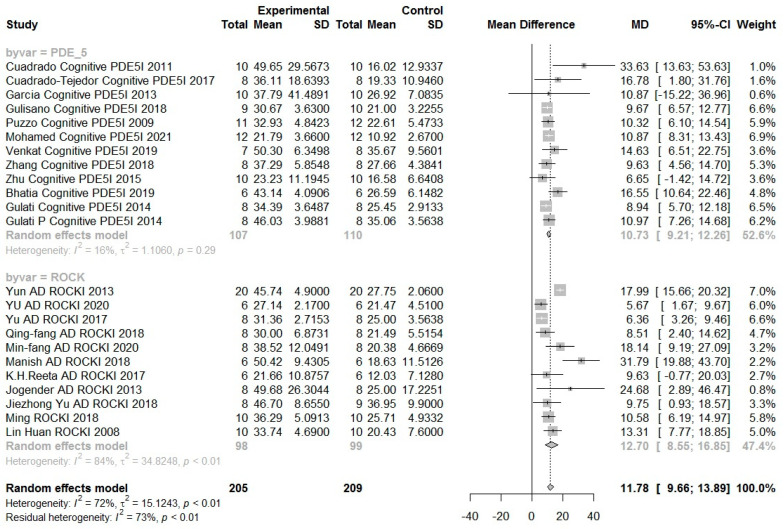
In the forest plot, the difference between the control group and the experimental group of each subgroup was analyzed by mean difference (MD). Each subgroup is located at the top and bottom with PDE-5 inhibitors and ROCK inhibitors. Because of the high heterogeneity, each group was analyzed by dividing it into subgroups.

Considering that the scales of all studies are not the same and the heterogeneity is high, the analysis was divided into standardized mean difference (SMD, Figure 5) and mean difference (MD, Figure 6).

Mice, who received all treatments, had an average of 1.83% SMD (95% CI 1.43–2.24, *I*^2^ 64%) more cognitive improvement than before treatment. Among them, mice treated with PDE-5 inhibitors had 1.80% SMD (95% CI 1.25–2.34 *I*^2^ 61%) cognitive improvement before treatment, while mice treated with ROCK inhibitors had 1.87% SMD (95% CI 1.25–2.49 *I*^2^ 66%) cognitive improvement than before treatment. The biggest SMD improvement among publications with ROCK inhibitor treatment was found in the report of Yun AD ROCKI 2013, showing a difference of 4.69% (95% CI 3.44–5.94) SMD. Conversely, the smallest SMD difference was 0.97% (95% CI 0.26–2.19), as reported by K.H. Reeta AD ROCKI 2017. Among the reports receiving PDE-5 inhibitor treatment, the most obvious difference in SMD was 3.28% (95% CI 1.98–4.57), which was reported by Mohamed Cognitive PDE5I 2021. On the other hand, the PDE-5 inhibitor treatment with the smallest SMD difference was Garcia Cognitive PDE5I 2013, which reported 0.35% (95% CI 0.54–1.23).

Mice, who received all treatments, had an average of 11.78% MD (95% CI 9.66–13.89, *I*^2^ 73%) more cognitive improvement. Among them, mice treated with PDE-5 inhibitors had 10.73% MD (95% CI 9.21–12.26 *I*^2^ 16%) cognitive improvement, while mice treated with ROCK inhibitors had 12.70% MD (95% CI 8.55–16.85 *I*^2^ 84%) cognitive improvement. The biggest MD improvement noted among publications on ROCK inhibitor treatment was in the report Cuadrado Cognitive PDE5I 2011, showing a difference of 33.63% (95% CI 13.63–53.63) MD. Conversely, the least MD difference was 6.65% (95% CI −1.42–14.72), which was reported by Zhu Cognitive PDE5I 2015. Among the reports on PDE-5 inhibitor treatment, the most obvious difference in MD was 31.79% (95% CI 19.88–43.70), as reported by Manish AD ROCKI 2018. On the other hand, the PDE5I treatment with the smallest MD difference was YU AD ROCKI 2020, which reported an improvement of 5.67% (95% CI 1.67–9.67).

## 7. Conclusions

Dementia, which causes cognitive impairment, is one of the main causes of death and affects the families of many patients globally. The main causes of dementia are AD and VD, and its treatment is difficult. Many mechanism studies on the treatment of dementia have been performed. We focused on studies of ROCK and PDE-5. ROCK and PDE-5 contribute to AD deterioration through PS1 mutation and tau hyperphosphorylation, respectively; cause damage to the brain due to hypoxia, induced by decreased cerebral blood flow due to vasoconstriction; and contribute to increased pro-inflammatory marker levels and immune cell migration. Therefore, ROCK and PDE-5 inhibitors are receiving significant attention as pharmacological treatments for dementia. Improvement of the cGMP pathway and an increase in the cognitive function of mice following ROCK or PDE-5 inhibition were confirmed in animal and in vitro experiments. In addition, the MWM-TSTQ (%) results of animal models were compared based on the SMD and MD through meta-analysis, confirming that both ROCK inhibitors and PDE-5 inhibitors helped improve cognitive function.

Therefore, the results of this analysis expect synergistic treatment effects for the combined administration of both drugs. Both ROCK and PDE-5 inhibitors showed good effects on improving cognitive impairment, and many factors share both mechanisms. ROCK suppresses eNOS and PDE-5 suppresses NO, leading to a downward adjustment of NO/cGMP. And they share several factors that can affect neuroinflammatory responses, such as MCP-1, NF-kB, and VCAM-1. So we conclude that the combined administration of both inhibitors is worth studying in anticipation of the treatment effect of synergy.

## Figures and Tables

**Table 1:** Newcastle-Ottawa quality assessment scale cohort studies.

Study	Model	Selection				Comparability		Outcome		
ROCK Inhibitor		1	2	3	4	1a	1b	1	2	3
Yun AD ROCKI 2013 [101]	AD	*	*	*	*	*		*	*	*
YU AD ROCKI 2020 [102]	AD	*	*	*	*	*	*	*	*	*
Yu AD ROCKI 2017 [103]	AD	*	*	*	*	*	*	*	*	*
Qing-fang AD ROCKI 2018 [104]	AD	*	*	*	*	*	*	*	*	*
Min-fang AD ROCKI 2020 [105]	AD	*	*	*	*	*	*	*		*
Manish AD ROCKI 2018 [106]	AD	*	*	*	*	*	*	*	*	*
K.H. Reeta AD ROCKI 2017 [107]	AD	*	*	*	*	*	*	*	*	*
Jogender AD ROCKI 2013 [108]	AD	*	*	*	*	*		*	*	*
Jiezhong Yu AD ROCKI 2018 [109]	AD	*	*	*	*	*	*	*	*	*
Ming ROCKI 2018 [110]	VD	*	*	*	*	*	*	*		*
Lin Huan ROCKI 2008 [111]	VD			*	*	*	*	*	*	*
**PDE-5 inhibitor**										
Cuadrado Cognitive PDE5I 2011 [112]	AD		*	*	*	*	*	*	*	*
Cuadrado-Tejedor Cognitive PDE5I 2017 [113]	AD		*	*	*	*	*	*	*	*
Garcia Cognitive PDE5I 2013 [114]	AD		*	*	*	*	*	*	*	*
Gulisano Cognitive PDE5I 2018 [115]	AD		*	*	*	*	*	*		*
Puzzo Cognitive PDE5I 2009 [116]	AD		*	*	*	*	*	*		*
Mohamed Cognitive PDE5I 2021 [117]	AD		*	*	*	*	*	*	*	*
Venkat Cognitive PDE5I 2019 [118]	AD		*	*	*	*	*	*	*	*
Zhang Cognitive PDE5I 2018 [119]	AD		*	*	*	*	*	*		*
Zhu Cognitive PDE5I 2015 [120]	AD		*	*	*	*		*	*	*
Bhatia Cognitive PDE5I 2019 [121]	VD		*	*	*	*	*	*		*
Gulati Cognitive PDE5I 2014 [122]	VD		*	*	*	*	*	*		*
Gulati P Cognitive PDE5I 2014 [123]	VD		*	*	*	*	*	*		*

* Indication means that the publication corresponds to the part of the Newcastle-Ottawa quality assessment. AD: Alzheimer’s disease, VD: vascular dementia, PDE5I: phosphodiesterase 5 inhibitor, ROCKI: rho-associated kinase inhibitor.

**Table 2:** Baseline characters for the control group and experimental groups extracted from the selected publications.

Study	Model		Sex	Age	Treatment Drug Injection	Injected Volume
ROCK Inhibitor	Control (Disease)	Experimental (Treatment)				
Yun AD ROCKI 2013	Ab1–42	Fasudil	Male	N.R	stereotaxic, left lateral ventricle	10 mg/kg
YU AD ROCKI 2020	APP/PS1	*Ganoderma lucidum* Triterpenoids (ROCKI)	Male	3 months	gavage	1.4 g/kg
Yu AD ROCKI 2017	APP/PS1	Fasudil	N.R	N.R	I.P	25 mg/kg/day
Qing-fang AD ROCKI 2018	APP/PS1	Fasudil	N.R	8 months	I.P	25 mg/kg/day
Min-fang AD ROCKI 2020	APP/PS1	Fasudil	N.R	8 months	N.R	25 mg/kg/day
Manish AD ROCKI 2018	ICV-STZ	Fasudil	N.R	N.R	I.C.V	3 mg/kg in 10 μL
K.H.Reeta AD ROCKI 2017	ICV-STZ	*Edaravone* (ROCKI)	Male	N.R	I.C.V	10 mg/kg
Jogender AD ROCKI 2013	ICV-STZ	*Clitoria ternatea* (ROCKI)	Male	N.R	I.C.V	500 mg/kg
Jiezhong Yu AD ROCKI 2018	APP/PS1	Fasudil	Male	8 months	I.C.V	25 mg/kg/day
Ming ROCKI 2018	BCAO	Y-27632	Male	N.R	I.P	10 mg/kg
Lin Huan ROCKI 2008	BCAL	Fasudil	Male	N.R	I.P	10 mg/kg
**PDE-5 inhibitor**						
Cuadrado Cognitive PDE5I 2011	Tg2576	Sildenafil	Female	14–16 months	I.P	15 mg/kg/day
Cuadrado-Tejedor Cognitive PDE5I 2017	Tg2576	CM-414 (PDE5i)	Female	14–16 months	I.P	40 mg/kg/day
Garcia Cognitive PDE5I 2013	J20	Sildenafil	Both	3 months	oral gavage	15 mg/kg
Gulisano Cognitive PDE5I 2018	APPswe	Vardenafil	Both	9–10 months	I.P	0.01 mg/kg
Puzzo Cognitive PDE5I 2009	APP/PS1	Sildenafil, Tadalafil	Both	3 months	I.P	3 mg/kg/day
Mohamed Cognitive PDE5I 2021	ICV-STZ	Tadalafil	Male	2 months	I.C.V	20 mg/kg/day
Venkat Cognitive PDE5I 2019	Cholesterol crystal	Sildenafil	Male	16–18 months	Internal carotid artery	2 mg/kg/day
Zhang Cognitive PDE5I 2018	Multiple micro infarction	KJH-1002 (PDE5i)	Male	9 weeks	gavage	20 mg/kg
Zhu Cognitive PDE5I 2015	APP/PS1	Sildenafil	Male	7 months	I.P	6 mg/kg
Bhatia Cognitive PDE5I 2019	BCAO	Tadalafil	Both	8–9 weeks	oral	10 mg/kg
Gulati Cognitive PDE5I 2014	BCAO	Tadalafil	Male	N. R	N. R	20 mg/kg
Gulati P Cognitive PDE5I 2014	BCAO	Tadalafil	Male	N. R	N. R	20 mg/kg

BCAO: bilateral carotid artery ligation, ICV: intracerebral vascular, ICV-STZ: intracerebral vascular -streptozotocin, I.P: intraperitoneal, N.R: no record, PDE5I: phosphodiesterase 5 inhibitor, PS1: presenilin 1, ROCKI: rho-associated kinase inhibitor.

**Table 3:** Time spent in the target quadrant (%) during the Morris water maze investigation extracted from each publication. The mean and standard deviation values and the populations of the experimental and control groups are presented.

Study	Model	Control (Disease)			Experimental (Treatment)		
ROCK Inhibitor		Mean (%)	SD (%)	Number	Mean (%)	SD (%)	Number
Yun AD ROCKI 2013	AD	27.75	2.06	20	45.74	4.90	20
YU AD ROCKI 2020	AD	21.47	4.51	6	27.14	2.17	6
Yu AD ROCKI 2017	AD	25.00	1.26	8	31.36	0.96	8
Qing-fang AD ROCKI 2018	AD	21.49	1.95	8	30.00	2.43	8
Min-fang AD ROCKI 2020	AD	20.38	1.65	8	38.52	4.26	8
Manish AD ROCKI 2018	AD	18.63	4.70	6	50.42	3.85	6
K.H.Reeta AD ROCKI 2017	AD	12.03	2.91	6	21.66	4.44	6
Jogender AD ROCKI 2013	AD	25.00	6.09	8	49.68	9.30	8
Jiezhong Yu AD ROCKI 2018	AD	36.95	3.30	9	46.70	3.06	8
Ming ROCKI 2018	VD	25.71	1.56	10	36.29	1.61	10
Lin Huan ROCKI 2008	VD	20.43	7.60	10	33.74	4.69	10
**PDE-5 inhibitor**							
Cuadrado Cognitive PDE5I 2011	AD	16.02	4.09	10	49.65	9.35	10
Cuadrado-Tejedor Cognitive PDE5I 2017	AD	19.33	3.87	8	36.11	6.59	8
Garcia Cognitive PDE5I 2013	AD	26.92	2.24	10	37.79	13.12	10
Gulisano Cognitive PDE5I 2018	AD	21.00	1.02	10	30.67	1.21	9
Puzzo Cognitive PDE5I 2009	AD	22.61	1.58	12	32.93	1.46	11
Mohamed Cognitive PDE5I 2021	AD	10.92	2.67	12	21.79	3.66	12
Venkat Cognitive PDE5I 2019	AD	35.67	3.38	8	50.30	2.40	7
Zhang Cognitive PDE5I 2018	AD	27.66	1.55	8	37.29	2.07	8
Zhu Cognitive PDE5I 2015	AD	16.58	2.10	10	23.23	3.54	10
Bhatia Cognitive PDE5I 2019	VD	26.59	2.51	6	43.14	1.67	6
Gulati Cognitive PDE5I 2014	VD	25.45	1.03	8	34.39	1.29	8
Gulati P Cognitive PDE5I 2014	VD	35.06	1.26	8	46.03	1.41	8

AD: Alzheimer’s disease, VD: vascular dementia, PDE5I: phosphodiesterase 5 inhibitor, ROCKI: rho-associated kinase inhibitor, SD: standard deviation.

## Data Availability

No new data were created or analyzed in this study. Data sharing does not apply to this article.
